# Optimal energy management of multi-carrier energy system considering uncertainty in renewable generation

**DOI:** 10.1038/s41598-025-10404-4

**Published:** 2025-07-17

**Authors:** Ankit Garg, K. R. Niazi, Shubham Tiwari, Sachin Sharma, Tanuj Rawat

**Affiliations:** 1 Department of Electrical Engineering, Malaviya National Institute of Technology, Jaipur, India; 2 Department of AFE, IIASA, Laxenburg, Vienna Austria; 3 Department of Electrical and Electronics Engineering, Manipal Institute of Technology, Manipal Academy of Higher Education, Manipal, Karnataka India; 4 GE Vernova, Noida, India

**Keywords:** Multi-carrier energy system, PEV, Optimization, Economic, Generation flexibility, Demand response, Energy management, Engineering, Energy grids and networks

## Abstract

This paper presents a structured approach for the efficient operation of multi-carrier energy systems under the uncertainty of renewable energy sources. As the penetration of wind and solar energy increases, managing the resulting variability becomes critical to maintaining both economic efficiency and operational flexibility. To address this, a two-stage multi objective optimization framework is proposed. In the first stage, the objective is to minimize daily operational costs while incorporating the uncertain behavior of renewables using a scenario-based stochastic approach. The second stage focuses on simultaneously enhancing system flexibility by maximizing the available capacities for both electrical and thermal energy generation and improving green house emissions. To evaluate system adaptability, two performance indicators are introduced: the Average Energy Generation Flexibility Index (AEGFI) and the Average Thermal Generation Flexibility Index (ATGFI). The optimization model is solved using the Modified Water Evaporation algorithm. Sensitivity analyses are also conducted to explore the effects of fluctuations in gas and electricity prices on system performance. The proposed model is applied to a generalized multi-carrier energy system. Simulation results demonstrate significant improvements in flexibility, with AEGFI and ATGFI increasing by 27.43% and 39.91%, respectively. Overall, the framework offers a comprehensive solution to balance cost-effectiveness and flexibility in energy systems with high shares of renewables.

## Introduction

In the Conventional power system, the electrical and gas energy distribution systems have typically been operated and functioning independently without taking into account their interrelationships. Due to a lack of coordination between multi-energy carriers reduces efficiency, raises operating costs and degrades system performance. Population growth has boosted natural resource demand in recent decades. Global energy consumption relies on fossil fuels. However, fossil fuels emit greenhouse gases and pollute^1,2^. Thus, a quick fix is needed. Addressing energy needs consumption worldwide, energy system modeling and planning focus. Researchers suggest microgrids (MGs) near load centers connect them with locally generated renewable energy (RES) to boost economic and traditional system sustainability^3,4^. In the contemporary age of energy systems, the landscape of energy management has been drastically modified as a result of the incorporation of renewable energy sources and the rise of multi-carrier energy systems^5,6^. When it comes to ensuring that energy supply is both efficient and reliable, multi-carrier energy systems, which are characterized by the coexistence of several energy carriers such as electricity, gas, heat and hydrogen, present potential as well as obstacles^7,8^. One of the most important aspects of the efficient operation of these intricate systems is the control of energy flows, particularly in light of the inherent uncertainties that are present in the generation of energy from renewable sources^9,10^. Most of the electrical energy in prior systems is produced by large-scale power machinery. Distributed power sources are considered to be small resources, loads or energy storage with explicit rules to address these challenges^11^. Examples of energy supplies that are smaller in scale compared to commonly used energy sources include solar panels, electricity-generating turbines, combined power and heat storage systems, diesel generators and variable loads^12,13^. The simultaneous operation of various energy production and transmission systems, such as gas and electricity networks, has been discussed in connection with the energy hub. Investors in this sector are concerned about the viability of energy hubs given the presence of several hazards, which are further intensified by various energy carriers^14,15^. It has been implemented to reduce greenhouse gas emissions, enhance power generation and decrease pricing. Additional notable issues arising from the utilization of the energy hub encompass system adaptability and peak shaving^16,17^. The primary goals are to reduce greenhouse gas emissions and decrease the total expenses of an energy center. In order to accomplish these objectives, it is necessary to comply with particular limitations, including the computation of the present load, the duration of the load during periods of high and low demand, and the prediction of the uncertain power output of distributed generators, such as solar and wind turbines^18,19^.

Due to the fact that they are less expensive and less harmful to the environment, sources of clean energy, such as solar and wind power, are becoming increasingly important in multi-carrier energy systems. On the other hand, the processes involved in the generation of energy are subject to enormous uncertainty due to the fact that they are unexpected and intermittent^20–22^. System operators and coordinators encounter challenges due to swings in renewable energy generation, stemming from various stochastic factors such as variation in the seasons, temperature changes, and weather fluctuations^23,24^. Renewable energy sources like wind and solar power pose a difficulty due to their irregular and unexpected energy generation. Focusing on minimizing the variability in energy production has been a crucial study topic in energy management^25,26^. Considering this issue allows decision-makers to develop effective methods for optimizing energy systems while considering objectives such as reducing expenses, conserving energy and advancing environmental sustainability.

## Literature review

In^1^, researchers created a way to cut operating expenses while considering different objectives. Another study^27^ used alternative time frames to reduce energy system expenditures ahead of time. A approach for managing uncertainty in Integrative Energy System management was proposed in another research^12^. An evolutionary algorithm was used to explore how hybrid energy storage devices affect IES scheduling^16^. In another study^20^, a model was built to schedule energy usage in phases to balance operational costs, reliability and flexibility. Despite incorporating uncertain consumption and renewable energy, these studies ignored water systems. Renewable energy sources present new problems for energy system operation planning, needing flexibility to adapt^23,28^. This flexibility is crucial for reliable and cost-efficient management. Reference^29^ created a complete model for system design by utilizing mixed-integer linear programming (MILP) and accounting for the unpredictability of MCES. However, the potential for adjusting and optimizing thermal and electrical power production was not taken into account^30^. The MCES has the ability to store energy through a charging mode when there is excess power available^31^. The energy saved can be used during times of increased demand or when Renewable energy production falls below the predicted quantities.

**Table 1:** Comparison with existing literature work.

References	Methodology	Advantages	Disadvantages
^9^	Transactive energy management	Role of electrical and thermal energy storage is considered. Effect of DR is considered.	Effect of uncertainties are ignored. Wind power curtailment is not considered.
^22^	CVaR method stochastic programming Monte-carlo simulation benders decomposition technique is used	Maximize the expected profit and minimize the total customers consumption costs. Effect of electric DR is considered Uncertainties of load is considered	Effect of thermal DR is not considered. Uncertainties of EV, RES and prices are not considered. DG Outage contingency is not considered.
^23^	Rolling horizon method MILP is used for day-ahead scheduling. MIQP is used for local energy scheduling.	Three types of energy sources (i.e. Electricity, heat and gas) are considered. Both Demand response of microgrids and electricity sharing among microgrids is consi- deredin the proposed strategy.	Uncertainties of load and RES is ignored. Effect of Cooling energy system is not considered. Flexibility issues are not considered.
^24^	Cooperative based transactive energy management	Load and demand uncertainties are considered. Effect of DR is considered.	Flexibility issues are not considered. RES uncertainties are not considered.
^27^	IGDT approach single stage framework is used	Effect of both electrical and thermal DR is considered. Load and price uncertainties are considered. Electrical flexibility is considered.	Curtailment of wind power is not considered. RES uncertainties is ignored. Thermal Flexibility is not considered.
^32^	Deterministic approach single objective framework	Electrical and thermal storage are considered. Cooperative energy management is used. Load uncertainties are considered	Effect of electric and thermal flexibility is not considered. RES and price uncertainties are ignored.
^33^	Stochastic model probabilistic scenario based approach	Minimize electrical and natural gas energy cost Price uncertainties are considered Electric Flexibility is considered	Uncertainties of load and RES are ignored. Effect of thermal flexibility is not considered. Role of DR is not considered.
This study	Two stage model stochastic optimization	Effect of electric and thermal flexibility is considered. DR effect is considered. RES uncertainties is considered.Second stage is multi-objective and solved using weighted sum approach.	

A four-objective assessment framework was proposed in reference^34^ to assess the effects of energy storage systems (ESS). However, the study did not directly examine the versatility of generation and Consumers. The proposed methodology took into account the probabilistic characteristics of wind energy in order to fulfill the necessary power demands. Nevertheless, this analysis disregarded demand-side flexibilities.

The multistage stochastic model in Ref.^35^ examines the impact of demand-side programmes on MCES schedule planning using a binary genetic algorithm. The study^36^ investigated the enduring effects of energy storage devices and demand response (DR) systems on the planning of operation for the MCES. This multi-objective methodology aims to minimize operating expenses and environmental issues concurrently. Author did not study the flexibility of generation for electrical and thermal portions. Decreasing spinning reserve greatly impacts generation flexibility^37,38^. In the last few years multi-carrier energy systems are playing a vital role in the energy systems. In^26^ optimal scheduling of multi carrier energy systems is proposed with the AC power flow constraints. In this study, the author considers energy storage systems such as electrical and heat storage systems. However, it ignores the effect of wind power curtailment and issues of flexibility. In^39^ author introduces a two-stage robust optimization model for integrated energy hubs, addressing uncertainties in load demand, renewable generation, and market prices. It evaluates system performance through detailed simulations and shows improved flexibility and resilience in energy dispatch. A Two stage stochastic model has been proposed in^29^ by using bender decomposition strategy. This study also considers electrical demand response and thermal demand response in the absence of renewable uncertainties. For the optimum planning of MCES, a scenario based algorithm is proposed in the absence of electric vehicle and demand response. In^23^ robust optimisation is proposed for the MCES considering the uncertainty of electric vehicle. It ignored the effect of electrical, thermal demand response and RES uncertainty. Different types of energy storage systems are used for the energy management of MCES in the presence of demand response and electric vehicle while ignoring the effect of uncertainty of RES. Two stage stochastic model for the MCES considering electrical and heating systems has been proposed. Although, it ignores the effect of cooling system and uncertainty. In^10^, author proposed a model for energy management of MCES having electrical, heat and ice system to examine the effect of demand and uncertainty of electric vehicle. In^40^ author presents a robust energy management framework for multi-carrier systems using scenario-based stochastic optimization. It emphasizes the role of electric vehicles and demand response in mitigating uncertainty. In^32^, author has proposed the stochastic framework for energy management of MCES considering the electrical demand response and uncertainty in the load. The main aim of this objective is to minimize the operational cost. Although this study ignores the effect of thermal demand response, EV uncertainty and renewable uncertainty. To overcome this effect of demand response and EV uncertainty the author has proposed multi carrier framework in^25,41^. However it ignores the effect of RES uncertainty and load uncertainty. Author has discussed exchange of energy between different energy carriers in presence of electrical and thermal storage systems. While it ignores the effect of demand response and uncertainty. Binary coded genetic algorithm is used for energy management of MCES. It mainly considers the operation cost of grid and boiler but it ignores the effect of electrical and thermal demand response and effect of uncertainty. In^19^ Author has discussed the optimal planning of MCES considering electrical demand response and EV uncertainty. Although in this study Author ignores the RES uncertainty and thermal demand response. Stochastic optimization has been proposed in^13^ for the minimization of operational cost in the presence electrical, thermal demand response and EV uncertainty, load certainty. However in this study author didn’t consider the RES uncertainty and curtailment of wind power. Author has proposed the robust optimization for the energy management of MCES. In this study author mainly considers the electrical and thermal demand response in the presence of load uncertainty. Although it ignores the RES uncertainty and price uncertainty. From the literature most of the study has considered the EV uncertainty, load uncertainty and ignore the effect of RES uncertainty. From the literature DR methods provides cost saving capability by transferring energy use from peak to off-peak time. But due to the uncoordinated demand management peak load increases during off peak hours. which would require additional local generation to meet demand of MCES. This decreases the electrical and thermal generation flexibility. To overcome these issues a two stage optimization approach has been proposed. The optimization problem in this work is solved using MWEA algorithm^42^.

In this study, a stochastic two stage optimization is proposed to improve generation and demand-side flexibility.This concept uniformizes load profiles through coordinated DR procedures and prevents peak loads. This boosts MCES versatility and promotes RES incorporation in transmission networks.

The Contribution of this paper is given as follows:

1. A Two stage coordinated operation scheme for the multi-carrier energy system is proposed. The first stage aims at minimizing operation cost and the second stage aims at optimizing flexibility. Inclusion of the second stage enable MCES to handle un-expected changes. Second stage is multi-objective and it is solved using weighted sum approach.
2. The flexibility of the MCES is evaluated on the basis of two index, Average Energy Generation Flexibility Index (AEGFI) and Average Thermal Generation Flexibility Index (ATGFI).
3. A scenario-based method is implemented to incorporate uncertain effect of renewables.
4. Sensitivity analysis to investigate impact of gas and electricity price and efficiency if energy storage system is presented.

## Modelling of the system

### Modeling of electrical and thermal DR

The simulation involves implementing demand response programs for both electrical and thermal aspects based on models represented by Eqs. (1–4). Equation (1) specifically outlines the involvement of the Multi-carrier Energy System (MCES) in shiftable demand response (DR) systems, where the adjustments in power levels, whether increased or decreased, must balance out. The limitations for moving the periodic load are determined by Eqs. (2 and 3). Equation (4) plays a crucial role in determining the direction of these adjustments, whether pushing power levels upwards or downwards^33^.

**Fig. 1:**
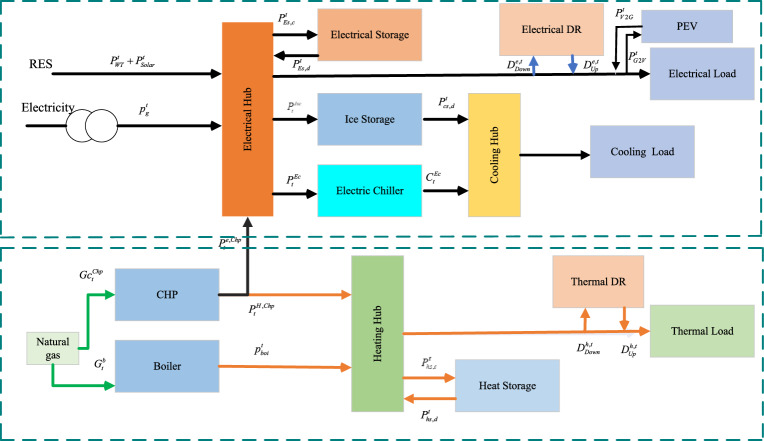
Architecture of the system.

1 $$\begin{aligned} & \sum _{t}D_{t}^{i,Up}=\sum _{t}D_{t}^{i,Down} \end{aligned}$$

2 $$\begin{aligned} & 0\le D_{t}^{i,Down}\le ML^{i,Down}l_{t}^{i} \gamma _{t}^{i,Down} \end{aligned}$$

3 $$\begin{aligned} & 0\le D_{t}^{i,Up}\le ML^{i,Up}l_{t}^{i} \gamma _{t}^{i,Up} \end{aligned}$$

4 $$\begin{aligned} & 0\le \gamma _{t}^{i,Up}+\gamma _{t}^{i,Down}\le 1 \end{aligned}$$

### Energy storage systems

Equations (5 and 6) set the boundaries for how much power can be used to charge or discharge energy in the system. The State of Charge (SoC) for storage systems is described in Eq. (7), indicating the level of energy stored. The allowable range for the Range of energy stored is determined by Eq.(8). Equation (9) determines the appropriate mode, either charging or discharging, for the system at a given time “t”. Equation (10) asserts that the final energy stored in the systems of storage must be equal to the starting energy output. In this formulation, the index i belonging to the set of systems for storing electricity, heat and cooling is denoted by e, h and c respectively, representing the different types of storage systems^25^.

5 $$\begin{aligned} & 0\le P_{t}^{i,Ch}\le P_{Max}^{i,Ch}q_{t}^{i,Ch} \end{aligned}$$

6 $$\begin{aligned} & 0\le P_{t}^{i,Dch}\le P_{Max}^{i,Dch}q_{t}^{i,Dch} \end{aligned}$$

7 $$Ess_{t}^{i} = Ess_{t}^{{i - 1}} + \left( {P_{t}^{{i,Ch}} \eta ^{{i,Ch}} } \right) - \left( {\frac{{P_{t}^{{i,Dch}} }}{{\eta ^{{i,Dch}} }}} \right)$$

8 $$\begin{aligned} & Ess_{Min}^{i}\le Ess_{t}^{i}\le Ess_{Max}^{i} \end{aligned}$$

9 $$\begin{aligned} & 0\le q_{t}^{i,Ch}+q_{t}^{i,Dch}\le 1 \end{aligned}$$

10 $$\begin{aligned} & Ess_{1}^{i}=Ess_{24}^{i} \end{aligned}$$

### Uncertainty modelling of RES

The nature of the RESs is rather probabilistic. The uncertainty of RESs should be taken into account while modeling a realistic optimization task. Probability density functions (PDF) are used to produce the scenarios in this work. There are a lot of scenarios in the scenario generation process, can be produced. Solution accuracy is increased by taking into account a large number of possibilities. The sun radiation scenarios are managed using a stochastic technique. A probability distribution function (PDF) for solar radiations is created to generate scenarios.

In this study, a large set of scenarios was initially generated using stochastic sampling techniques based on probability density functions for solar irradiance (Beta distribution) and wind speed (Weibull distribution). To manage the computational complexity, a scenario reduction technique was applied. This method selects a subset of scenarios that maintain the statistical properties of the original set by minimizing the overall deviation in probability space. Specifically, the reduction aimed to preserve key statistical features–such as mean and variance–while limiting the number of representative scenarios to four. This reduced set was found to be sufficient, as additional simulations showed minimal differences in operational cost and flexibility indexes (AEGFI and ATGFI), typically within a 2–3% range. This confirms that the reduced scenario set still offers accurate insights while significantly lowering the computational burden.

#### Modelling of solar generation

Modelling of solar radiations is described by the beta probability distribution function^14^.

11 $$\begin{aligned} & f_{PDF}(S)=\frac{\Gamma (p+q)}{\Gamma (p)\Gamma (q))} S^{p-1} (1-S)^{q-1}; S\varepsilon (0,1) \end{aligned}$$

12 $$\begin{aligned} & q=(1-\phi ) (\frac{\phi (1+\phi )}{\sigma ^{2}}-1); \, \, \, p=\frac{\phi q}{1-\phi } \end{aligned}$$

where p and q are the parameters of beta PDF.

#### Modelling of wind speed

The Weibull probability density function (PDF) is typically used to represent the change of wind speed. Equation (14) provides the PDF function that connects the wind speed and WT output power^14^.

13 $$P_{{WT,S}}^{t} = \left\{ {\begin{array}{*{20}c} {0,} & {{\text{if}}\;\theta _{\omega }^{t} \le \theta _{{min}} \;or\;\theta _{\omega }^{t} \ge \theta _{{max}} } \\ {P_{{WT}}^{r} \frac{{\theta _{\omega }^{t} - \theta _{{min}}^{t} }}{{\theta _{{\omega ,r}}^{t} - \theta _{{min}}^{t} }}} & {{\text{if}}\;\theta _{{min}} \le \theta _{\omega }^{t} \le \theta _{r} } \\ {P_{{WT}}^{r} } & {{\text{if}}\;\theta _{r} \le \theta _{\omega }^{t} \le \theta _{{max}} } \\ \end{array} } \right.$$

**Fig. 2:**
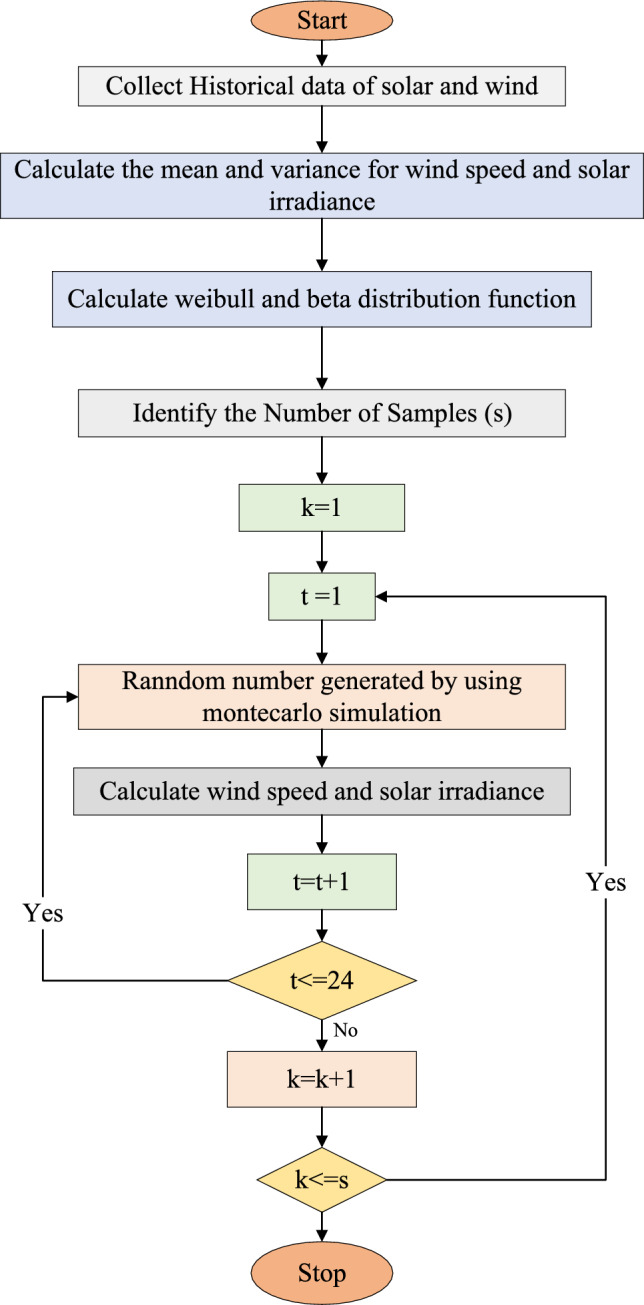
Flowchart for scenario generation and reduction.

**Fig. 3:**
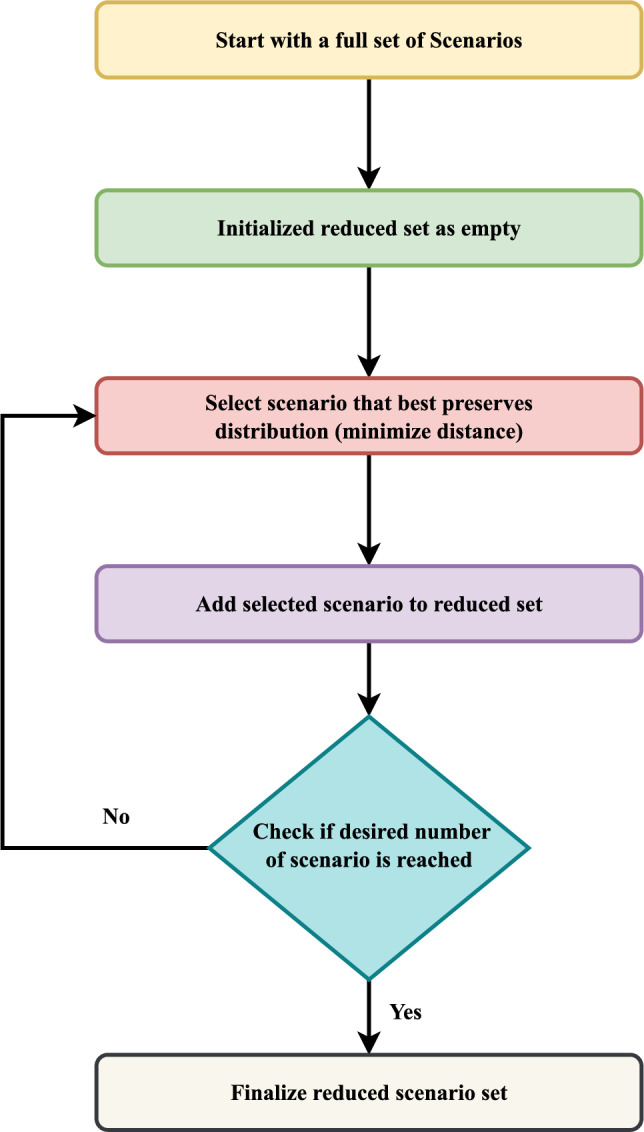
Flowchart for scenario reduction algorithm.

14 $$f_{{PDF}} (\nu ) = {\text{ }}\frac{k}{\psi }\left( {\frac{\nu }{\psi }} \right)^{{k - 1}} e^{{ - \left( {\frac{\nu }{\psi }} \right)^{k} }}$$

15 $$k = {\text{ }}\left( {\frac{\sigma }{\psi }} \right)^{{ - 1.086}}$$

16 $$\begin{aligned} \psi= & \frac{\phi }{\Gamma (1+\frac{1}{k})} \end{aligned}$$

### Plug in EV

The limitations of electric vehicle charging and discharging capacity have been illustrated in Eqs. (17 and 18). Equation (19) is used to figure out the charging status or condition of electric vehicles at a specific time, denoted as t. Equation (20) establishes the permissible states of charge ranges. The energy consumed while in transit is computed using Eq. (21). Equation (22) ultimately ascertains whether the device is in the charging or discharging state^32^.

17 $$\begin{aligned} & 0\le P_{t,u}^{Pev,Ch}\le P_{Max}^{Pev,Ch}I_{t,u}^{Pev,Ch} \end{aligned}$$

18 $$\begin{aligned} & 0\le P_{t,u}^{Pev,Dch}\le P_{Max}^{Pev,Dch}I_{t,u}^{Pev,Dch} \end{aligned}$$

19 $$Soc_{{t,u}}^{{Pev}} = Soc_{{t - 1,u}}^{{Pev}} + \left( {P_{{t,u}}^{{Pev,Ch}} \eta _{u}^{{Pev,Ch}} } \right) - \left( {\frac{{P_{{t,u}}^{{Pev,Dch}} }}{{\eta _{u}^{{Pev,Dch}} }}} \right) - P_{{t,u}}^{{Pev,Tr}} {\text{ }}$$

20 $$\begin{aligned} & Soc_{Min}^{Pev}\le Soc_{t,u}^{Pev}\le Soc_{Max}^{Pev} \end{aligned}$$

21 $$\begin{aligned} & P_{t,u}^{Pev,Tr}=\Delta Td_{t,u}^{Pev}\times \eta _{u}^{Pev} \end{aligned}$$

22 $$\begin{aligned} & 0\le I_{t,u}^{Pev,Ch}+I_{t,u}^{Pev,Dch}\le 1 \end{aligned}$$

### Power balance

The power balance indicates that MCES must maintain consistent electrical generation and load throughout time slots. Equation (23) details the SIES electrical power balance. Additionally, Eq. (24) determines CHP unit electricity generation. Power input to ISC is restricted by Eq. (25). Equation (26) summarizes electric chiller electricity consumption limits. Furthermore, Eq. (27) presents the minimum and maximum limits for buying or selling power to and from the main power grid^14^.

23 $$\begin{aligned} & \begin{aligned}&P_{t}^{Grid}+\sum _{s}\rho _{s}(P_{t,s}^{Solar}+P_{t,s}^{Wind})+P_{t}^{e,Chp}+D_{t}^{e,Down}+P_{t}^{e,Dch}+\sum _{u}P_{t,u}^{Pev,Dch}=\sum _{s}\rho _{s}L_{t,s}^{e}+D_{t}^{e,Up}\\ &\quad +P_{t}^{e,Ch}+\sum _{u}P_{t,u}^{Pev,Ch}+P_{t}^{Isc}+P_{t}^{Ec} \end{aligned} \end{aligned}$$

24 $$\begin{aligned} & P_{t}^{e,Chp}=Gc_{t}^{Chp}LCV\eta ^{e,Chp} \end{aligned}$$

25 $$\begin{aligned} & 0\le P_{t}^{Isc}\le P_{Isc}^{Max} \end{aligned}$$

26 $$\begin{aligned} & 0\le P_{t}^{Ec}\le P_{Ec}^{Max} \end{aligned}$$

27 $$\begin{aligned} & -K_{Max}^{Grid}\le P_{t}^{Grid}\le K_{Max}^{Grid} \end{aligned}$$

### Heat balance

Equation (28) indicates equal heat generation and thermal demands for MCES at each time unit. Boiler and CHP heat generation is calculated using (29) and (30). Equations (31 and 32) specify the input gas ranges for the boiler and CHP. In addition, Eq. (33) restricts input heat to AC. Boiler thermal power production is limited by Eq. (34) The permitted ranges for supplied natural gas are shown in (35) and (36). Finally, the heating system limits are in (37)^14^.

28 $$\begin{aligned} & P_{t}^{H,Chp}+H_{t}^{b}+D_{t}^{H,Down}+P_{t}^{H,Dch}= l_{t}^{h}+D_{t}^{H,Up}+P_{t}^{H,Ch}+H_{t}^{ac} \end{aligned}$$

29 $$\begin{aligned} & H_{t}^{b}=G_{t}^{b}LCV\eta ^{b} \end{aligned}$$

30 $$\begin{aligned} & P_{t}^{H,Chp}=G_{t}^{Chp}LCV\eta ^{Chp} \end{aligned}$$

31 $$\begin{aligned} & 0\le G_{t}^{b}\le G_{Max}^{b} \end{aligned}$$

32 $$\begin{aligned} & 0\le G_{t}^{Chp}\le G_{Max}^{Chp} \end{aligned}$$

33 $$\begin{aligned} & 0\le H_{t}^{ac}\le H_{Max}^{ac} \end{aligned}$$

34 $$\begin{aligned} & 0\le H_{t}^{b}\le H_{Max}^{b} \end{aligned}$$

35 $$\begin{aligned} & 0\le P_{t}^{Gas}\le P_{Max}^{Gas} \end{aligned}$$

36 $$\begin{aligned} & P_{t}^{Gas}=G_{t}^{b}+G_{t}^{Chp} \end{aligned}$$

37 $$\begin{aligned} & 0\le P_{t}^{H,Chp}+H_{t}^{b}+P_{t}^{H,Dch}-P_{t}^{H,Ch}-H_{t}^{ac}\le P_{Max}^{H} \end{aligned}$$

### Cooling balance

Modelling of Cooling Power balance is illustrated in Eqs. (38−41)^17^.

38 $$\begin{aligned} & C_{t}^{Ec}+C_{t}^{ac}+P_{t}^{C,Dch}=l_{t}^{c} \end{aligned}$$

39 $$\begin{aligned} & P_{t}^{C,Ch}=P_{t}^{Isc}COP^{Isc} \end{aligned}$$

40 $$\begin{aligned} & C_{t}^{ac}=H_{t}^{ac}COP^{ac} \end{aligned}$$

41 $$\begin{aligned} & C_{t}^{Ec}=P_{t}^{Ec}COP^{Ec} \end{aligned}$$

### Key assumptions and limitation of the proposed system

These include the use of scenario-based stochastic modeling for solar and wind uncertainties, fixed demand and price profiles per scenario, and ideal operation of storage systems without aging or losses. The system is assumed to be fully coordinated, with no network-level constraints or communication delays considered. The model does not incorporate network-level power flow constraints. Limitations such as the absence of cooling systems, simplified DR behavior and the exclusion of power-to-gas technologies are now explicitly discussed.

## Two stage structure for flexible generation and demand

In the proposed work two stage optimization model has been developed. Where first stage is used for minimization of the operation cost and the objective of the second stage is used for enhance the generation flexibility. The methodology combines factors such as operational expenses, the versatility of electricity and heat generation and the adjustability of demand-side components to create a comprehensive scheduling mechanism. The MCES carries out scheduling to identify the optimal operating costs, represented as C*. This C* value is then passed to the next step, where it is used to adjust the primary schedule to improve generation flexibility.

### First stage of the framework

This section encompasses the economic functioning of MCES. The MCES minimizes daily operation expenses by considering power expenditures, upstream signal pricing and load flexibility.

42 $$\begin{aligned} & f_{1}= Min C=C^{Grid}+C^{Chp}+C^{b}+C^{Edr}+C^{Hdr} \end{aligned}$$

43 $$\begin{aligned} & C^{Grid}=\sum _{t}\sum _{s}\rho _{s}P_{t}^{Grid}\mu _{t}^{e} \end{aligned}$$

44 $$\begin{aligned} & C^{Chp}= \sum _{t}G_{t}^{Chp}\pi _{t}^{g} \end{aligned}$$

45 $$\begin{aligned} & C^{b}= \sum _{t}G_{t}^{b}\pi _{t}^{g} \end{aligned}$$

46 $$\begin{aligned} & C^{Edr}= \sum _{t}\left( C_{dr}^{e,Down}D_{t}^{e,Down}+C_{dr}^{e,Up}D_{t}^{e,Up} \right) \end{aligned}$$

47 $$\begin{aligned} & C^{Hdr}= \sum _{t}\left( C_{dr}^{h,Down}D_{t}^{h,Down}+C_{dr}^{h,Up}D_{t}^{h,Up} \right) \end{aligned}$$

**Algorithm 1:**
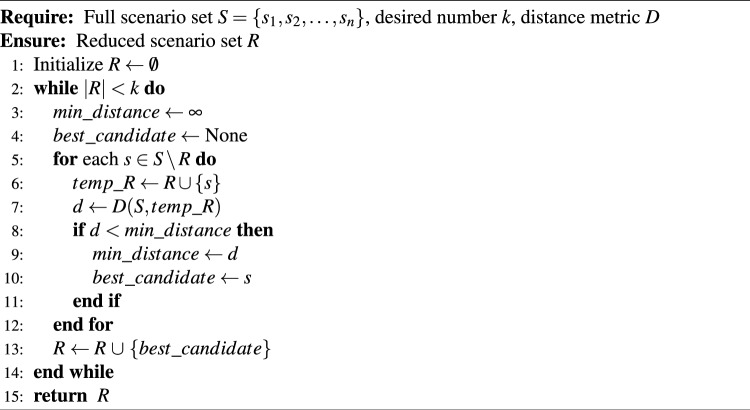
Forward selection-based scenario reduction

### Second stage of the model

The variability of renewable energy sources (RES) and fluctuating electricity demands pose new challenges for managing multi energy systems effectively. Flexibility in these systems refers to their ability to adapt to changes. This stage is multi-objective and it is solved using weighted sum approach. This flexibility can be achieved through various methods, which are divided into solutions related to generation and those related to managing demand. This particular aspect focuses on solutions related to generation, aiming to increase both electricity and heat production simultaneously. By increasing the reserved capacity, the system gains more flexibility in generation. Therefore, efforts in this aspect aim to maximize the available capacity for both electricity and heat generation while meeting the required energy demands.

48 $$\begin{aligned} Em_{Net}^{t}= & Em_{CO_{2}}^{t}\, \, +Em_{SO_{x}}^{t}\, \, +Em_{NO_{x}}^{t} \end{aligned}$$

49 $$\begin{aligned} f_{2}= & Em_{Net}^{t}=Min\begin{Bmatrix}\left( Y_{e,CO_{2}}+ Y_{e,SO_{x}}+Y_{e,NO_{x}}\right) \left\{ \sum _{t}\sum _{m}P_{e,g}^{t} \right\} + \\ \left( Y_{DG,CO_{2}}+ Y_{DG,SO_{x}}+Y_{DG,NO_{x}} \right) \left\{ \sum _{t}\sum _{m}P_{DG}^{t} \right\} + \\ \left( Y_{NG,CO_{2}}+ Y_{NG,SO_{x}}+Y_{NG,NO_{x}} \right) \left\{ \sum _{t}\sum _{m}P_{g,NG}^{t} \right\} \end{Bmatrix} \end{aligned}$$

50 $$\begin{aligned} f_{3}= & Max.\sum _{t}[Es_{t}^{e}+(P_{Max.}^{e,Chp}-P_{t}^{e,Chp})]+\sum _{t}[Es_{t}^{h}+(P_{Max.}^{h,Chp}-P_{t}^{h,Chp})] \end{aligned}$$

**Fig. 4:**
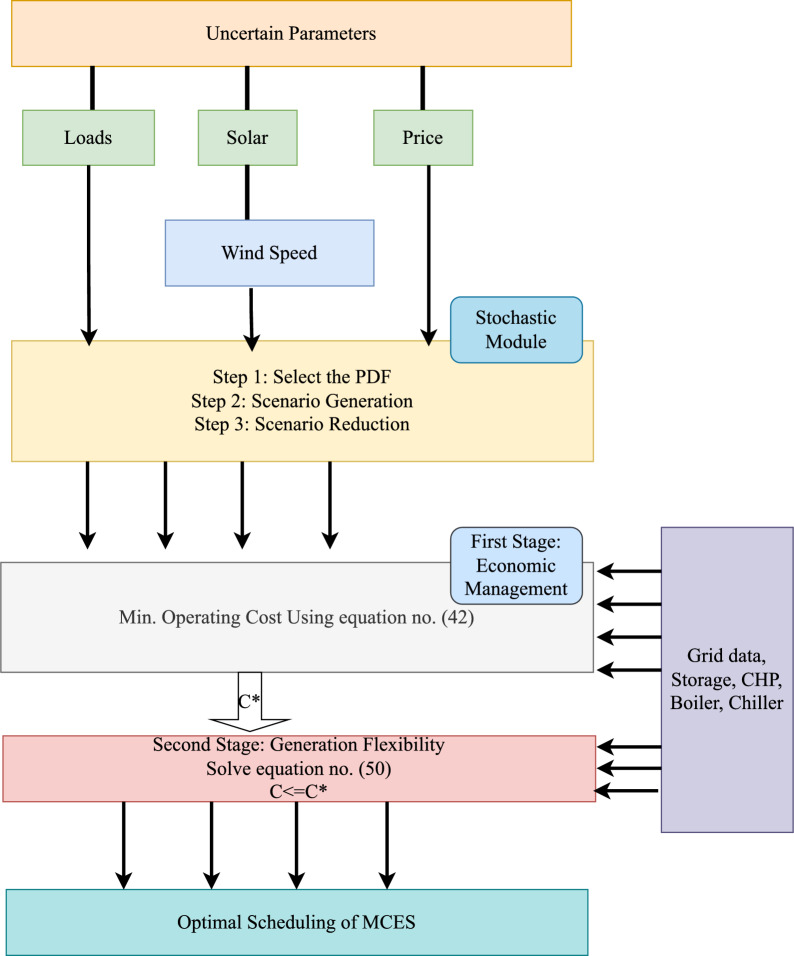
Block diagram for proposed two stage model.

### Modified water evaporation algorithm

**Fig. 5:**
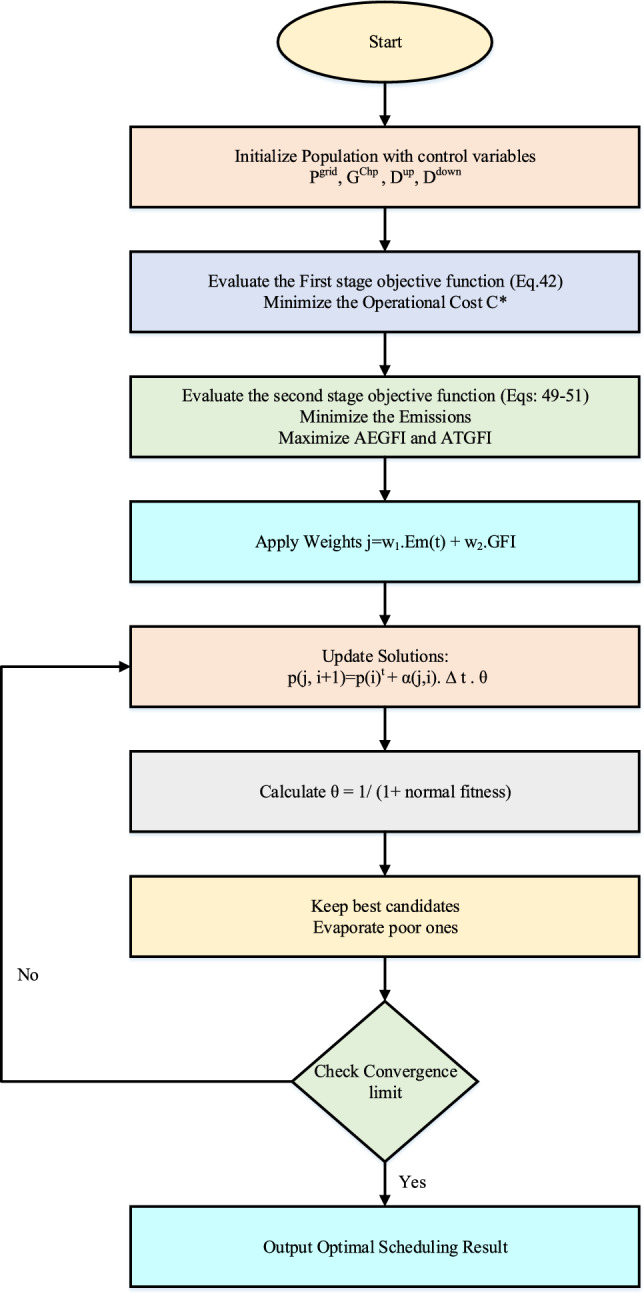
Block diagram for MWEA.

The Modified Water Evaporation Algorithm (MWEA) is used in this study as a metaheuristic optimization tool to solve the complex two-stage energy scheduling problem and designed to optimize multi-objective energy scheduling under uncertainty. It updates candidate solutions for control variables such as grid power purchase, CHP generation, energy storage, and demand response by mimicking the natural evaporation behavior of water molecules. The algorithm evaluates both economic (Eq. 42) and emission-based (Eq. 49–51) objectives using a weighted fitness function. A factor called $\theta$, calculated from the fitness, controls how much a solution changes. Candidates with better performance are retained, while poor ones are evaporated. This balance between exploration and exploitation enables the algorithm to efficiently search the solution space and converge to an optimal energy dispatch schedule under uncertain conditions. Inspired by the natural evaporation of water, it iteratively improves candidate solutions by simulating directional movement and selective retention of better-performing energy plans^42^. The algorithm effectively handles the non-linearity and uncertainty introduced by renewable sources and demand response. By updating solution vectors based on evaporation dynamics, MWEA explores a wide search space without getting trapped in local optima. Its adaptability makes it suitable for optimizing both economic and flexibility objectives. This ensures robust scheduling of distributed energy resources under uncertain operating conditions. Flowchart of the MWEA algorithm is represented in Fig. 5.

### Two stage weighted optimization

This study employs a two-stage, priority-driven optimization framework to address the multi-objective problem (MOP). The primary objective–minimizing the network’s operational cost–is prioritized and optimized in the first stage. In the second stage, the additional objectives, GHG emissions and Generation Flexibility Index (GFI), are concurrently minimized while ensuring compliance with the cost constraint derived from the initial stage.

The first stage focuses solely on minimizing operational costs, setting a cost threshold $C^{*}$. The second stage then maximizes the flexibility of the generation while ensuring that the total cost remains within $C\le C^{*}$. Trade-offs are managed through a multi-objective weighted function that incorporates two normalized terms: emissions and flexibility indices. The weight factors $\omega _{1}$ and $\omega _{2}$ guide the balance between environmental impact and system flexibility. To improve transparency, we now include a sensitivity range analysis for these weights (Table 3), showing how different combinations influence the Pareto frontier. This provides justification for the selected values and illustrates the model’s responsiveness to different planning priorities.

51 $$\begin{aligned} Min\left\{ w_{1}\left| \frac{f_{2}-f_{2}^{*}}{f_{2}^{*}}\right| +w_{2}\left| \frac{f_{3}-f_{3}^{*}}{f_{3}^{*}} \right| \right\} \end{aligned}$$

### Flexibility indexes

AEGFI and ATGFI are critical indexes for assessing the flexibility of electrical and thermal energy generation, subsequently. These indexes quantify a system’s capacity to adjust its energy generation in response to fluctuations in demand or variations in operational conditions, providing valuable insight into the adaptability and efficiency of energy management frameworks. AEGFI indicates the adaptability of the electrical generation system. When the AEGFI value increases, it represents improved ability to efficiently modify output in response to demand variations, thereby improving system reliability and cost-effectiveness. Likewise, the ATGFI assesses the adaptability of thermal generation, where a higher index indicates a superior response to fluctuations in heating. These indexes are key characteristic for systems that manage both electrical and thermal energy, such as combined heat and power systems. Flexibility indexes are defined by Eqs. (52 and 53).

52 $$\begin{aligned} AEGFI= & \frac{1}{n_{t}}\sum _{t=1}^{n_{t}}SOC_{t}^{e,ESS}+ (P_{Max}^{Chp}-P_{t}^{Chp}) \end{aligned}$$

53 $$\begin{aligned} ATGFI= & \frac{1}{n_{t}}\sum _{t=1}^{n_{t}}SOC_{t}^{h,ESS}+ (H_{Max}^{Chp}-H_{t}^{Chp}) \end{aligned}$$

The efficacy, reliability and superiority of the recommended Two stagestructure were demonstrated by two case studies conducted on the MCES. The formulation of AEGFI and ATGFI is intended to measure the generation-side flexibility by capturing two key components: the available energy stored in the system and the unused capacity of the CHP unit. The sum of the storage level and the reserve margin of the CHP at each hour provides a direct estimate of how much the system can adapt to unexpected changes in load or renewable generation. Averaging this value over the scheduling horizon gives a consistent indicator of the system’s flexibility. This approach ensures that both stored energy and headroom in dispatchable generation are considered in evaluating the system’s real-time responsiveness.

The AEGFI and ATGFI indices are proposed to measure electrical and thermal flexibility in uncertain conditions. Unlike conventional metrics that focus only on reserve margins or static capacities, these indices consider the system’s real-time response using storage and demand-side resources across all scenarios. Their uniqueness lies in capturing average generation flexibility in a structured, scenario-based way. They are useful for assessing how well the system adapts to renewable variability without relying on cost-based optimization alone. However, a key limitation is that they rely on model-derived outputs and assume ideal control execution, which may not fully reflect real-world constraints such as communication delays or equipment degradation.

Case 1: The objective in this scenario is to decrease the total expenses for the following day by considering the utilization of sustainable energy sources and different energy storage technologies and programs for managing both electrical and thermal demand response.

Case 2: In this case MCES is using a recommended framework to enhance its operational efficiency. The focus is on improving operating costs, as well as the flexibility in thermal generation and electrical generation.

## Results and discussion

**Fig. 6:**
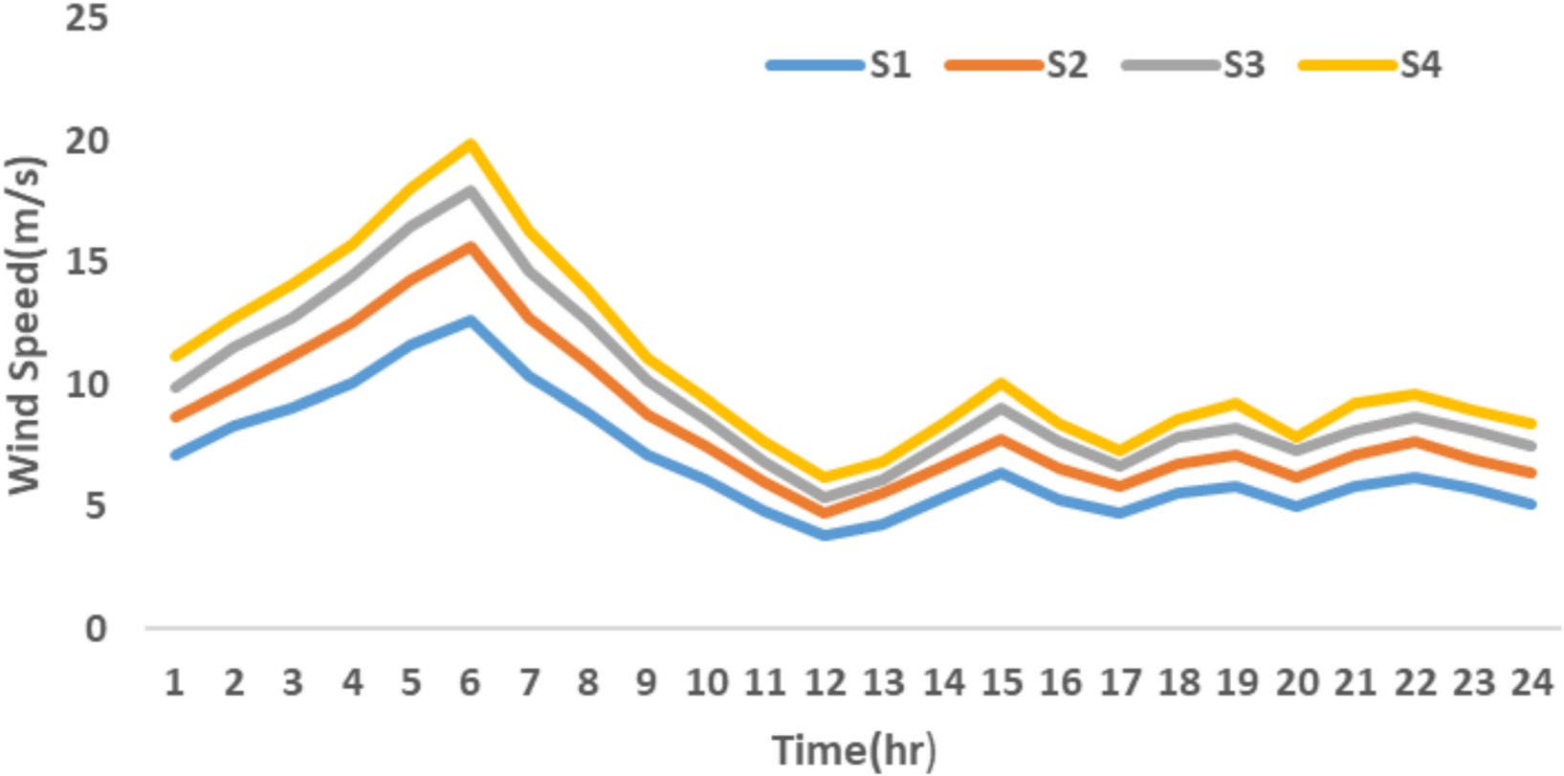
Wind speed for different scenario.

**Fig. 7:**
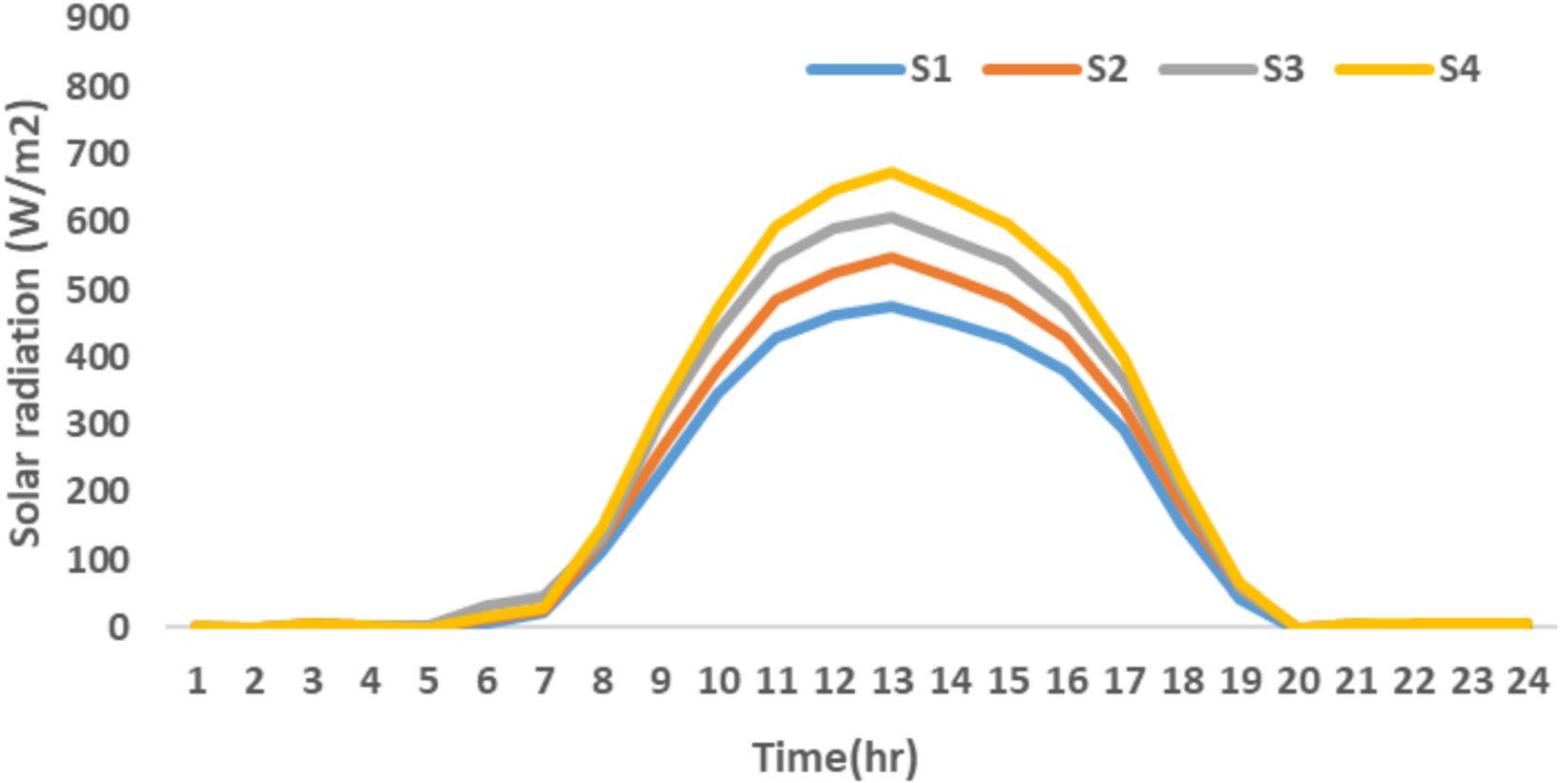
Solar irradiation for different scenario.

**Fig. 8:**
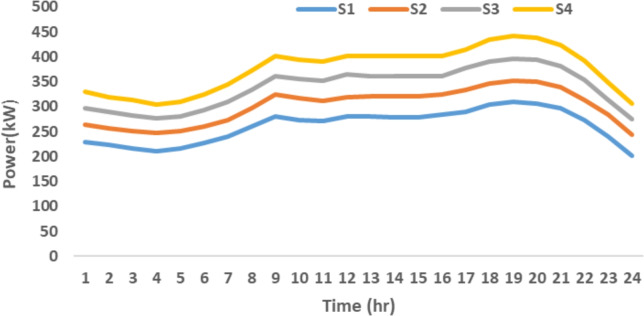
Load for different scenarios.

**Fig. 9:**
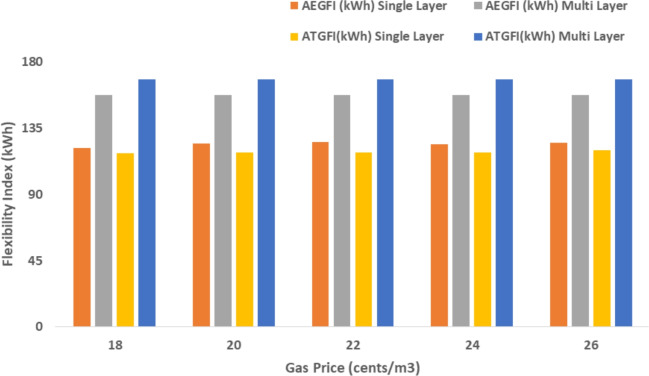
Price for different scenarios.

The proposed system is illustrated in Fig. 1 presents the system architecture, providing an overview of its structural components. Figure 2 depicts the flowchart for scenario generation and reduction, outlining the methodology for managing uncertainties. Scenario reduction algorithm is represented by Figs. 3 and 4 illustrates the block diagram of the proposed two-stage model, detailing the operational framework and optimization process (Fig. 5). Two case studies are modelled to demonstrate the proposed model. Figures 6, 7 and 8 shows the wind speed, solar irradiation and load for different scenarios respectively.The upper limit for energy transfers with the grid is assumed at 700 kW. The CHP unit functions with electrical and thermal efficiency of 35% and 45% respectively. The boiler has an efficiency of 80% and possesses a maximum heat production capacity of 20 kW. To address stochastic variability inherent in renewable energy output, demand and pricing Four scenarios are generated. Input data for the wind and solar is taken from^32,33,43^. CHP, Boiler and Energy storage input parameters are taken from^8,22,24^. Market price for different scenarios is represented by the Fig. 9. Table 1 provides a comparison with existing literature, highlighting the advancements and distinctions of the proposed approach. The Simulation findings of Table 2 indicates that AEGFI increases from 121.41 to 154.72 kWh and ATGFI increase from 118.23 to 165.42 kWh. By using the multi stage, there is a significant improvement in the flexibility index 27.43% and 39.91% for AEGFI and ATGFI respectively. Table 3 presents the Pareto solutions for the second-stage optimization, demonstrating the trade-offs among multiple objectives. Proposed model reduces the peak load and improves the load factor as shown in Table 4. Peak load in the multi stage model reduces from 457.31 to 398.74 kW which is decreased by 12.80%. Additionally this model enhances the load factor by 14.75%. Load profile for different case study is represented by Fig. 8. In Case Study-1 there is a new peak appears because of the lower price of the electricity during the off-peak time causing the shift a substantial portion of consumption to minimize operating costs. However By using the proposed model it smooths the load profile and enhances the performance of the system. The simulation findings reveal notable improvements in energy generation and flexibility when utilizing a multi-stage model. Specifically, the Average Energy Generation Flexibility Index (AEGFI) increases from 121.41 to 154.72 kWh. Additionally, the multi-stage framework enhances the Average thermal Generation Flexibility Index (ATGFI) from 118.23 to 165.42 kWh. To confirm the reliability of the reduced set, we compared outcomes such as operational cost and flexibility indices across different scenario counts. The results showed minimal deviation within 2–3%, demonstrating that the selected scenarios effectively capture system behaviour.

The second level of the framework, which emphasizes on enhancing generation flexibility, leads to a substantial improvement. AEGFI and ATGFI show a significant increase of 27.43% and 39.91%, respectively, compared to the results from case study 1. The multi-stage model greatly reduces peak energy usage. Energy usage is more steady in the second stage, improving capacity utilization and maximum demand. The model lowered peak load by 12.80% in the first scenario, which was 457.36 kW. Due to its hybrid min–max and max–min methods, the model boosted load factor by 14.76%. Table 4 shows that an uncoordinated Demand Response (DR) program can cause an off-peak. Case 1 has a peak load of 442.85 kW without DR programs and 457.36 kW with an uncoordinated program. Stressing that flattening the energy consumption profile reduces peak demands improves system adaptability.

**Table 2:** Comparison results of case study.

Case study	Cost ($)	AEGFI (kWh)	ATGFI (kWh)
Case-1	81231.39	121.41	118.23
Case-2	85875.69	154.72	165.42
Improvement (%)	5.71	27.43	39.91

**Table 3:** Pareto solutions for the second-stage optimization.

S. no.	$C^{*}$	$\omega _{1}$	$\omega _{2}$	Emission (kg)	Generation flexibility (kWh)
1	Operating cost = 85875.65 (y=1.02)	0.1	0.9	47823.65	165.354
2		0.2	0.8	47767.75	165.279
3		0.3	0.7	47731.37	165.245
4		0.4	0.6	47672.72	165.216
5		0.5	0.5	47646.48	165.190
6		0.6	0.4	47635.45	165.078
7		0.7	0.3	47535.28	164.610
8		0.8	0.2	47567.65	164.395
9		0.9	0.1	47823.67	165.354

**Table 4:** Features of load profile.

Cases	Peak (kW)	Load factor (%)
Base load	442.85	83.58
Case-1	457.31	81.42
Case-2	398.74	93.43
Improvement (%)	12.80	14.75

**Fig. 10:**
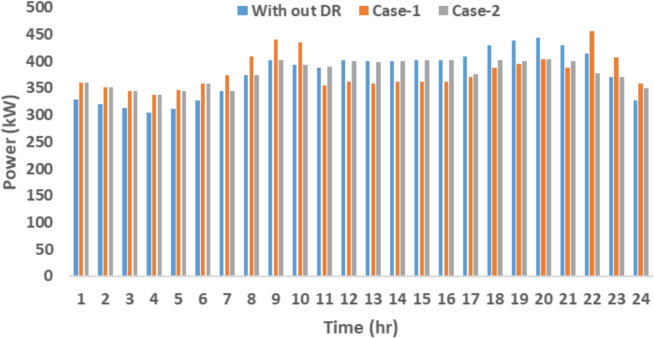
Load profile.

**Fig. 11:**
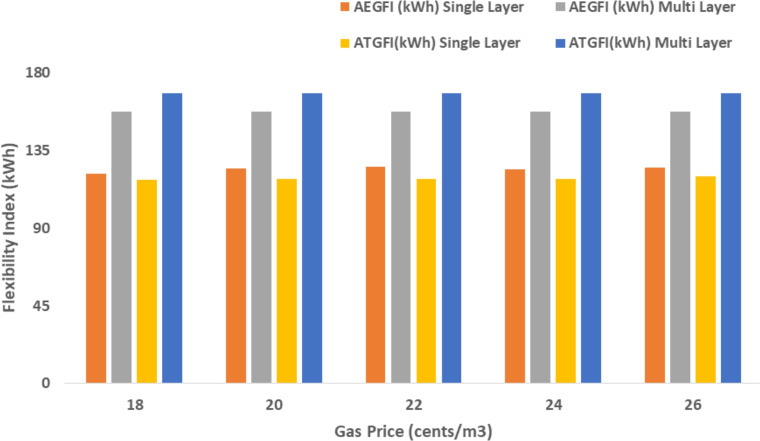
Sensitivity analysis on gas price.

**Fig. 12:**
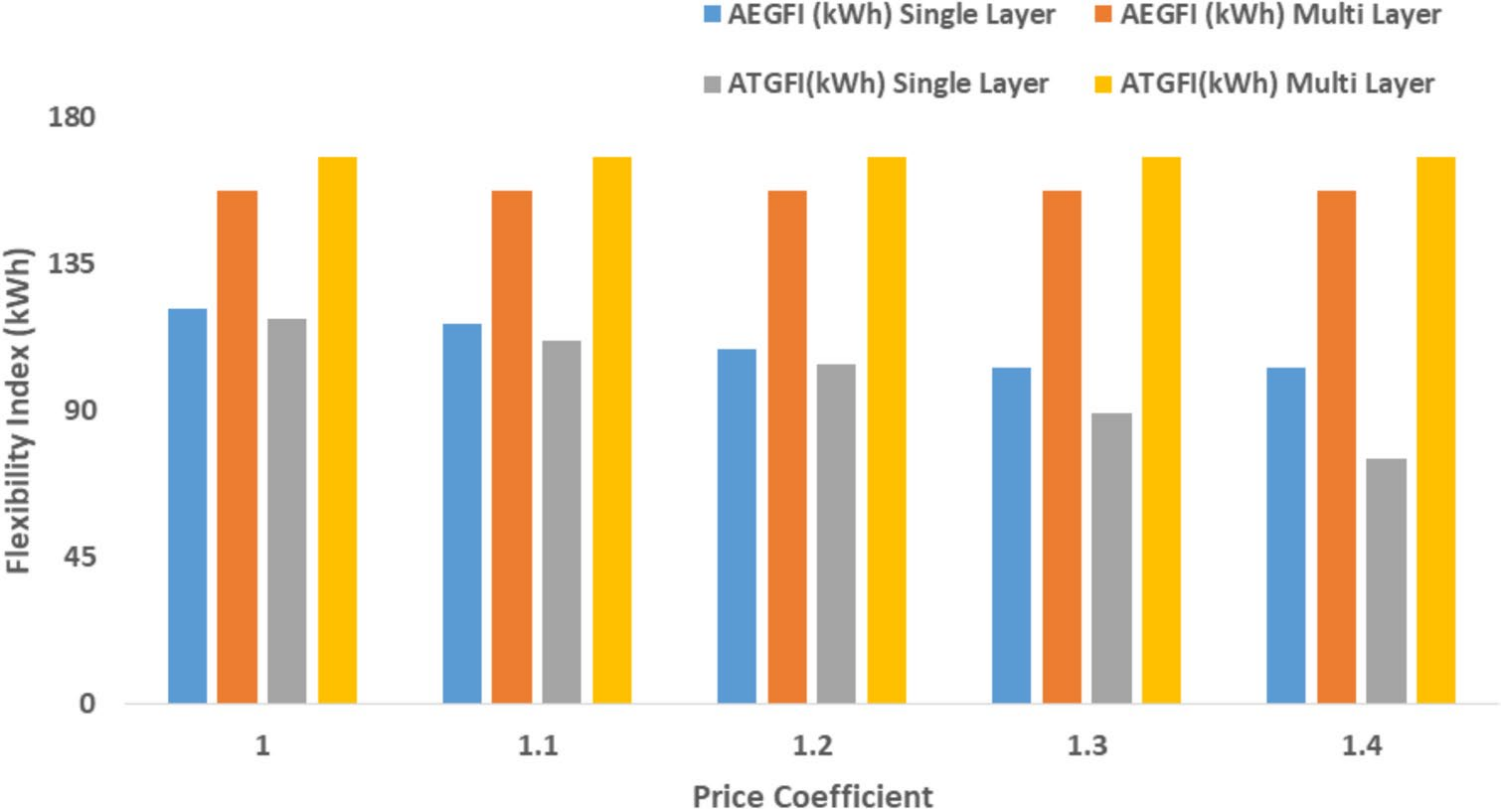
Sensitivity analysis on electricity price.

**Fig. 13:**
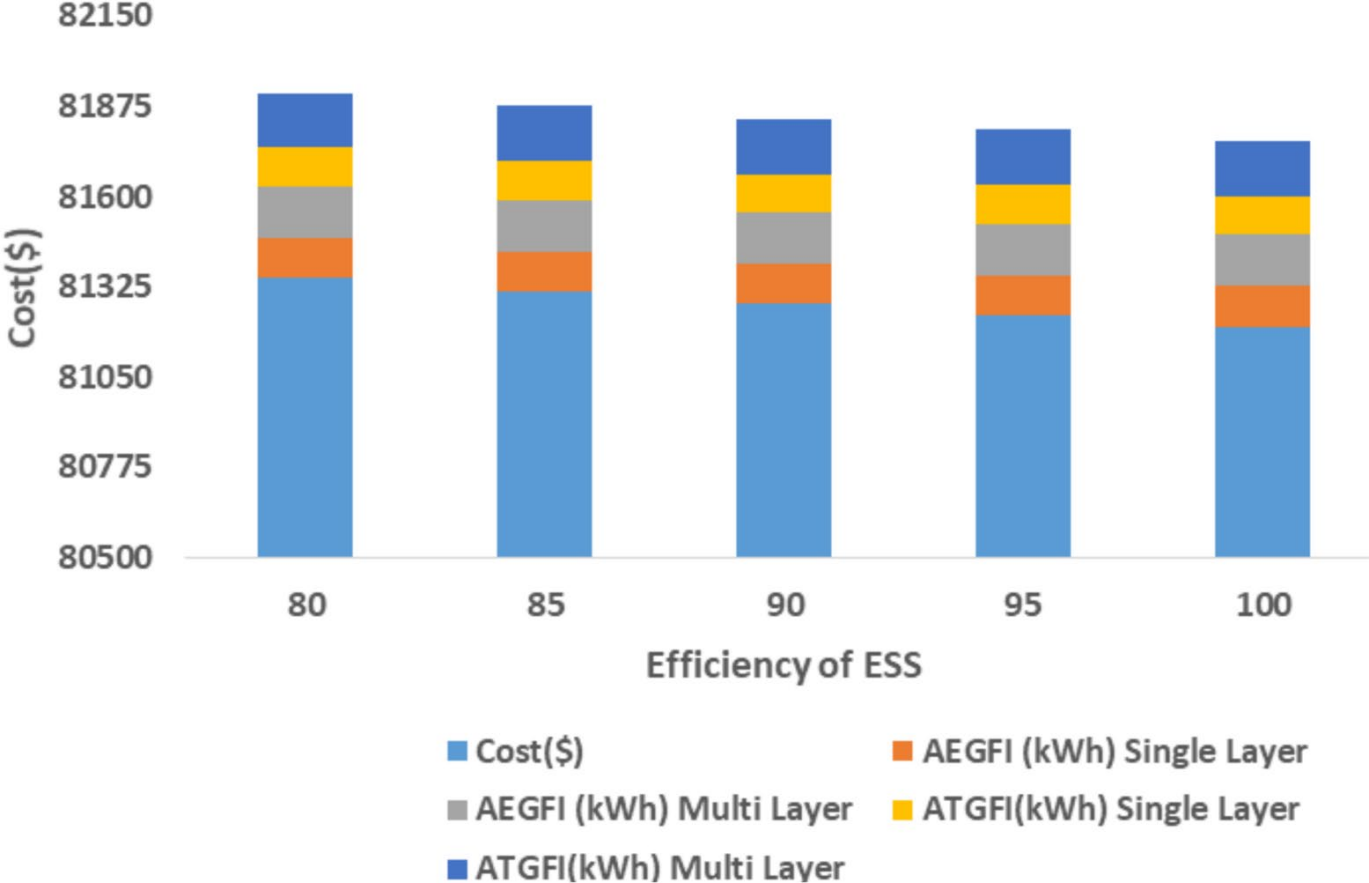
Sensitivity analysis on efficiency of ESS.

**Fig. 14:**
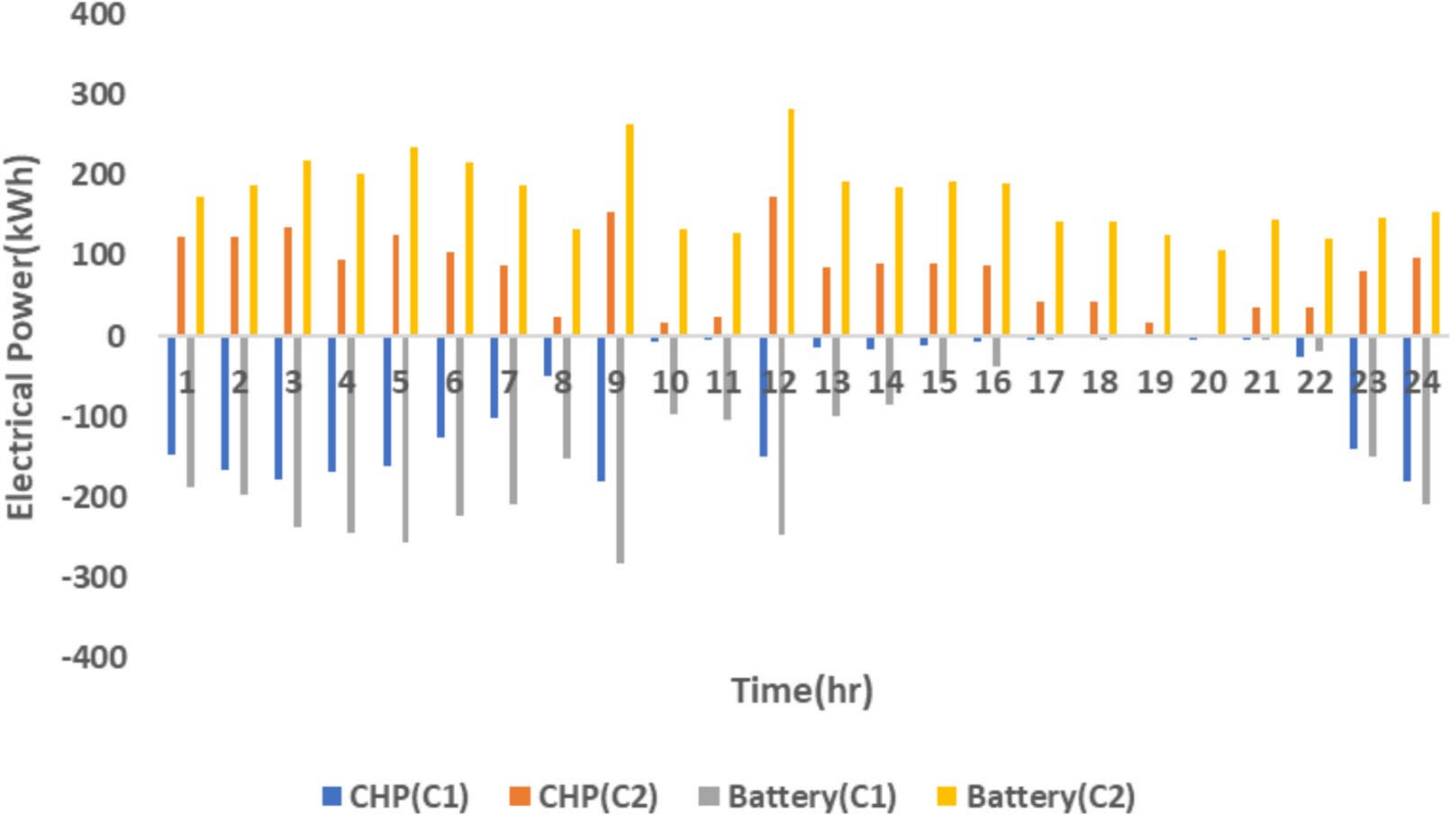
Flexibility of electricity generation.

**Fig. 15:**
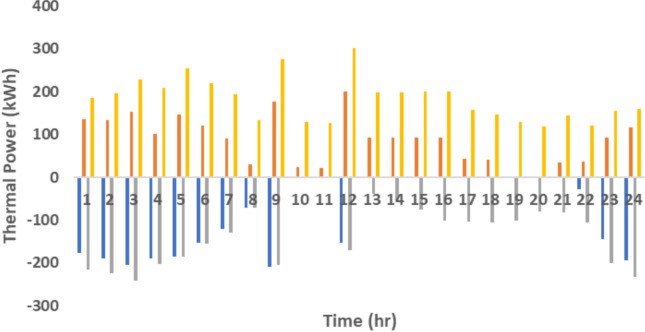
Flexibility of thermal generation.

## Sensitivity analysis

The following section presents a sensitivity analysis that showcases the effectiveness of the proposed multi-stage approach across many circumstances. Figure 11 presents the association between the price of gas and the effectiveness of the system. The sensitivity analysis highlights the influence of gas prices, electricity price coefficients, and energy storage efficiency on the system’s performance. As gas prices increase, both AEGFI and ATGFI show a noticeable improvement under the proposed multi-stage framework, indicating better utilization of flexible resources. In contrast, the single-objective approach reflects a decline in flexibility with price fluctuations, emphasizing the advantage of the two-stage model. Moreover, the proposed system maintains stable flexibility levels despite changes in electricity prices, demonstrating its robustness. When the efficiency of energy storage systems is increased, a clear reduction in operational cost is observed, along with a significant enhancement in generation flexibility. These results underline the effectiveness of the proposed model in adapting to variable market conditions while ensuring reliable and cost-effective energy management.

The price of gas has risen from 18 cents per cubic meter to 26 cents per cubic meter. The outcomes of the simulation are displayed in Fig. 10. The results indicated that the proposed technique offers greater flexibility in electrical and thermal generation compared to a single objective framework. The suggested model results in a minimum improvement of 25.43% for AEGFI and 40.03% for ATGFI, as shown in Fig. 10.

Simulation findings indicates that as the value of the coefficient increases (indicating an increase in energy price), both the AEGFI and ATGFI decline in the framework with only one goal as shown in Fig. 12. Figure 13 represnts the sesitivity analysis of EES. When the efficiency of ESS is increases there is a reduction in operation cost. Acoording to Fig. 11 the AEGFI and ATGFI improves atleast 27.80% and 41.62% correspondingly. Nevertheless, in the proposed approach, the AEGFI and ATGFI are unaffected by variations in electricity prices and are fixed at a consistent level of 157.51 kWh and 167.93 kWh, accordingly. The simulation findings indicate that the proposed strategy can enhance the AEGFI and ATGFI by 52.09% and 123.25% correspondingly, in different scenarios. This enhances spinning reservation during peak hours, helping the Multi-carrier energy System (MCES) manage Renewable Energy Source (RES) uncertainties. MCES uses most of its energy at these times to cut costs. On the other hand, the multi-stage model effectively evens out load patterns more efficiently. Figures 14 and 15 provide a comparison of electrical and thermal generation flexibility. The simulation findings indicate that Case 2 exhibits greater flexibility in both aspects. In Case 1, there is a lack of flexibility for MCES during the period from 18 to 21. Nevertheless, the suggested strategy guarantees a steady 100 kWh of flexibility for each time slot inside this specific time period. In addition, the suggested approach regularly outperforms Case 1 by providing over 100 kWh of thermal flexibility at each time interval. Significantly, Case 1 lacks thermal adaptability between the intervals of 10 and 11. Higher value of indexes, enable operators to better balance supply with demand fluctuations, enhancing system reliability, lowering operational costs and optimizing resource use. For end-users, these indexes contribute to greater stability and potential reductions in energy costs, along with a lowered risk of outages. Thus, MCES may better manage RES and load demand uncertainties with the suggested approach. MCES is more resilient in emergencies and can better control energy swings due to its flexibility.

The proposed two-stage framework highlights a clear trade-off between minimizing operational costs and maximizing system flexibility. The first stage focuses on minimizing cost, while the second stage improves flexibility without exceeding the initial cost limit. This setup helps evaluate how much flexibility can be added within budget constraints. Results show that a slight increase in cost leads to better use of storage and demand response. This trade-off supports more reliable and adaptive energy management.

## Conclusion

This work proposes a two stage optimization framework for optimal management of multi-carrier energy systems and solved by MWEA. The approach introduced in this framework brings together different sectors like electricity, heating, and cooling with the aim of improving the overall efficiency of the energy system. The first stage optimizes operation cost whereas the second stage enhances flexibility. The uncertainty in renewables in first stage is tackled using stochastic programming. Two metrics AEGFI and ATGFI are used to assess the flexibility of multi-carrier system. Moreover, a sensitivity analysis is presented to investigate impact of gas and electricity price on the operation of multi-carrier energy systems. The metrics show that the proposed two stage structure significantly improves the flexibility of the system. AEGFI and ATGFI are improved by 27.42% and 39.94% using the two stage structure as compared to single stage structure. The two stage structure appears highly relevant to real-world applications, particularly in smart grid environment where operation cost and flexibility are critical. In the future study we will extend this work in to the three stage framework by incorporating additional flexible resources, such as energy storage and power-to-gas systems. Future work will also focus on validating the model using real operational data from energy systems. This will help assess its performance under actual conditions and improve its practical relevance. We plan to use real load, generation and storage data to test the model’s accuracy and robustness. This step will strengthen its applicability for real-world deployments.

## Data Availability

The datasets used and/or analysed during the current study available from the corresponding author on reasonable request.

## List of symbols

$I^{Pev,Ch}/I^{Pev,Dch}$: Binary variables of PEV
*LCV*: Calorific value of gas
$\gamma _{t}^{i,Up}/\gamma _{t}^{i,Down}$: Binary variable for DR
$\theta _{w}$: Wind turbine speed
$\varphi _{\mu }$: Price variation in electricity
$C^{Chp}/C^{b}$: CHP/Boiler unit cost
$C^{Grid}$: Expense of procuring electricity from the upstream network
$D_{t}^{i,Up}/D_{t}^{i,Down}$: Shifted up/down power of DR
$ML^{i,Up}/ML^{i,Down}$: Maximum upward/downward shift for DR
$P_{t,u}^{Pev,Ch}/P_{t,u}^{Pev,Dch}$: Charging/discharging power of PEV
$P_{t}^{i,Ch}/P_{t}^{i,Dch}$: Charging/discharging power of energy storage
